# Adolescent depression beyond DSM definition: a network analysis

**DOI:** 10.1007/s00787-021-01908-1

**Published:** 2021-12-02

**Authors:** Pedro H. Manfro, Rivka B. Pereira, Martha Rosa, Hugo Cogo-Moreira, Helen L. Fisher, Brandon A. Kohrt, Valeria Mondelli, Christian Kieling

**Affiliations:** 1 Department of Psychiatry, School of Medicine, Universidade Federal do Rio Grande do Sul (UFRGS), Rua Ramiro Barcelos, 2350, 400N, Porto Alegre, RS 90035-903 Brazil; 2 Faculty of Teacher Education and Languages, Department of Education, ICT and Learning, Østfold University College, Halden, Norway; 3 Social, Genetic and Developmental Psychiatry Centre, Institute of Psychiatry, Psychology and Neuroscience, King’s College London, London, UK; 4 ESRC Centre for Society and Mental Health, King’s College London, London, UK; 5 Division of Global Mental Health, Department of Psychiatry, School of Medicine and Health Sciences, The George Washington University, Washington, DC USA; 6 Department of Psychological Medicine, Institute of Psychiatry, Psychology and Neuroscience, King’s College London, London, UK; 7 National Institute for Health Research Mental Health Biomedical Research Centre, South London and Maudsley NHS Foundation Trust and King’s College London, London, UK; 8 Child and Adolescent Psychiatry Division, Hospital de Clínicas de Porto Alegre (HCPA), Porto Alegre, RS Brazil

**Keywords:** Depressive symptoms, Adolescence, Psychometrics, Diagnostic and statistical manual of mental disorders, Patient outcome assessment

## Abstract

Calls for refining the understanding of depression beyond diagnostic criteria have been growing in recent years. We examined the prevalence and relevance of DSM and non-DSM depressive symptoms in two Brazilian school-based adolescent samples with two commonly used scales, the Patient Health Questionnaire (PHQ-A) and the Mood and Feelings Questionnaire (MFQ). We analyzed cross-sectional data from two similarly recruited samples of adolescents aged 14–16 years, as part of the Identifying Depression Early in Adolescence (IDEA) study in Brazil. We assessed dimensional depressive symptomatology using the PHQ-A in the first sample (*n* = 7720) and the MFQ in the second sample (*n* = 1070). We conducted network analyses to study symptom structure and centrality estimates of the two scales. Additionally, we compared centrality of items included (e.g., low mood, anhedonia) and not included in the DSM (e.g., low self-esteem, loneliness) in the MFQ. Sad mood and worthlessness items were the most central items in the network structure of the PHQ-A. In the MFQ sample, self-hatred and loneliness, two non-DSM features, were the most central items and DSM and non-DSM items in this scale formed a highly interconnected network of symptoms. Furthermore, analysis of the MFQ sample revealed DSM items not to be more frequent, severe or interconnected than non-DSM items, but rather part of a larger network of symptoms. A focus on symptoms might advance research on adolescent depression by enhancing our understanding of the disorder.

**Supplementary Information:**

The online version contains supplementary material available at 10.1007/s00787-021-01908-1.

## Introduction

Depressive disorders constitute a leading cause of health-related burden globally [1]. Depression tends to have its onset in adolescence [2] and is commonly chronic and recurrent, with lifetime cumulative prevalence estimates reaching 25% [3]. As a time of profound biopsychosocial changes, adolescence is an important period for the evaluation of mental health problems. Understanding unique characteristics of depression during this period can be crucial for alleviating its life-long repercussions, especially in low- and middle-income settings, where the majority of global youth live, but the minority of mental health research is conducted [4, 5].

The heterogenous nature of major depressive disorder (MDD) poses, however, multiple challenges towards this goal. The Diagnostic and Statistical Manual (5th edition; DSM-5) criteria for MDD among adolescents requires the presence of at least five out of nine possible symptoms, with one of those being low/irritable mood or anhedonia [6]. In adults, these criteria allow for over 200 symptom permutations that meet the current DSM diagnosis [7]—though such analysis has not been performed among adolescents, even greater heterogeneity would be theoretically expected given the additional criterion of irritability. MDD’s multitude of symptom profiles also impacts its understanding from neurobiological [8] and psychosocial [9] perspectives. Furthermore, a non-negligible portion of people receiving psychotherapeutic and/or pharmacological interventions—strategies usually employed following a one-size-fits-all approach to treatment—only partially benefit from them [10].

Suboptimal outcomes may in part stem from an over-focus on criteria that do not adequately consider patient priorities [11]. Items listed in the DSM may not fully capture the experience of living with depression in youth, as, historically, the DSM is a consensus-based operationalization of psychopathology [12] rather than an evidence- or data-driven one. Commonly used instruments for assessing depression dimensionally reflect such heterogeneity. Scales frequently reflect clinically significant symptoms that represent authors’ clinical views. For instance, the Children’s Depression Inventory (CDI) features items on self-deprecation, pessimism and loneliness that are not explicitly present in the DSM criteria but, much like its original adult version (the Beck Depression Inventory), reflects Beck’s cognitive model [13]. Conversely, the Children Depression Rating Scale (CDRS), based on the Hamilton Depression Rating Scale, prioritizes somatic symptoms [14], common among hospitalized patients with depression.

Despite the DSM’s binary approach to mental illness being undeniably relevant for decision-making in research and clinical settings [15], calls for better understanding of psychiatric symptomatology beyond categorical criteria have gained momentum in recent years. One promising avenue is the adoption of symptom-level, data-driven methods. The network framework [16] offers an alternative to the common cause model of disease, in which symptoms are caused by an underlying latent variable (e.g., low mood, anhedonia, concentration difficulties, insomnia and weight loss are all equally caused by “depression” in the same way a bacteria causes pneumonia). Alternatively, the network perspective considers symptoms as mutually reinforcing entities by focusing on symptoms rather than syndromes. In line with most of the research landscape [5], network analytic investigations of adolescent depression are also more commonly conducted in high-income settings [17–19], more specifically Western Educated Industrialized Rich and Democratic (WEIRD) populations with English-speaking samples [20]. Additionally, even though they are not mutually exclusive [21], most studies to date have examined depression symptoms either from a latent or a network approach.

Therefore, with the growing emphasis in the literature on understanding depression symptomatology beyond current DSM criteria and its interest in the generalizability of psychological findings [22], symptom-level analysis of MDD symptoms in adolescence is a promising avenue to move the field forward. Following from research in adult, clinical samples [23, 24], we hypothesize that symptoms of adolescent depression may be uniquely interconnected and may not follow strict DSM criteria. We here aimed to examine, in two school-based samples of Brazilian adolescents, the symptom structure of two commonly used dimensional depression scales.

## Methods

### Sample description

We analyzed cross-sectional data from two samples recruited from public state schools in Porto Alegre, Brazil. Both samples were composed of adolescents aged 14–16 years and both completed the same identification and sociodemographic questionnaire, but each had a different instrument to capture depressive symptomatology: one the Patient Health Questionnaire-Adolescent Version (PHQ-A)[25] the other, the Mood and Feelings Questionnaire (MFQ) [26]. The PHQ-A sample (*n* = 7720) was recruited from June 2018 to November 2019, while the MFQ sample (*n* = 1070) was recruited from August 2016 to December 2016. For the PHQ-A sample, 101 schools were visited; for the MFQ sample, 7 schools were visited. All schools in the MFQ sample were also visited for the PHQ-A sample. This report is part of the Identifying Depression Early in Adolescence (IDEA) study, a multi-national collaborative effort to advance the early identification of MDD in adolescents [27, 28]. As inclusion criteria for this study, adolescents had to be enrolled in grades 8–11 and be aged 14–16 years on the day of school recruitment. Participants and/or primary caregivers provided written dissent terms if they refused to participate and all data were coded to ensure anonymity in database handling. Independently of further inclusion in the IDEA study [28], trained psychologists and child psychiatrists contacted participants who reported suicidality, physical or sexual trauma for in-depth clinical evaluation and referral to appropriate care if needed in accordance with Brazilian legislation. The study was approved by the Brazilian National Ethics Committee (CAAE 50473015.9.0000.5327).

### Measures

#### Sociodemographic variables

Participants completed a questionnaire on age, gender, skin color, school information and parental age. Skin color followed the Brazilian Institute of Geography and Statistics (IBGE) census categorization as white, black, yellow, brown or indigenous. Adolescents also answered questions on variables which are part of a composite risk score for the risk of developing depression in adolescence, the Identifying Depression Early in Adolescence Risk Score [28, 29], though these were not included in the current analysis.

#### Patient Health Questionnaire-9—adolescent version (PHQ-A)

The PHQ-A is an adapted version of the Patient Health Questionnaire-9 **(**PHQ-9) for use with adolescents and is commonly employed as a screening tool in clinical and research settings [25]. The questionnaire consists of nine questions with Likert-type response options “none”, “several days”, “more than half the days” and “nearly every day”. The nine items were designed to represent the DSM-IV criteria for a major depressive episode. We performed the process of translation and cultural adaptation of the scale following the TRAPD (Translation, Review, Adjudication and Documentation) steps proposed for questionnaire translation and assessment [30]. In the PHQ-A sample, 3.1% of participants had missing values; therefore, we conducted multiple imputation, with no significant differences in the imputed sample and the whole sample regarding proportion of males/females, age, skin color, mean PHQ-A score or maltreatment history (Online Resources Table S1).

#### Mood and Feelings Questionnaire (MFQ)

The MFQ is a 33-item self-report questionnaire with three response options (“not true”, “sometimes true” and “true”) designed to assess mood symptomatology [26], recently translated and adapted to Brazilian Portuguese by our group [31]. It evaluates features included in the DSM criteria and those not explicitly included in the criteria (e.g., “I felt lonely”). We classified MFQ items as “non-DSM” according to previous studies [12, 23, 24]. Items 12 and 20 were categorized as social isolation; item 14 as easy crying; items 15, 22 and 28 as pessimism; items 23 and 25 as self-derogation; item 24 as self-accusation; item 26 as somatic complaints; item 27 as loneliness; item 30 as low-confidence and pessimism; item 31 as feelings of inadequacy/failure. To allow for comparable analysis between the PHQ-A and the MFQ, we combined DSM items using an “or” rule (e.g., items on reduced and increased appetite were combined to form one item reflecting the DSM A3 criterion; see Online Resources Table S2 for a full description). Since 5.1% of participants had missing values on the MFQ items we conducted multiple imputation, with no significant differences in the imputed sample and the whole sample regarding proportion of males/females, age, skin color, mean MFQ score or maltreatment history (Online Resources Table S1).

### Statistical analysis

We calculated mean and standard deviations (SD) for continuous variables, as well as frequencies and percentages for categorical variables. To evaluate possible school-level influence in questionnaire responses, we analyzed the intraclass correlation coefficient (ICC) by school for both samples [32]. We conducted Wilcoxon-Mann–Whitney tests to compare MFQ and PHQ-A median scores for boys and girls. We compared means, SD and centrality estimates between DSM and non-DSM features with permutation tests that compare the observed variables to a distribution of possible differences between groups.

#### Latent variable analysis

We used confirmatory factor analysis (CFA) to identify factor structure and dimensionality of the PHQ-A and the MFQ [21]. To test if PHQ-A and MFQ items could be reduced to a single “depressive symptomatology” factor, we tested unidimensional solutions. Model fit was evaluated based on traditional fit measures [33]: Comparative Fit Index (CFI) and Tucker-Lewis Index (TLI) ≥ 0.95; and root mean square error approximation (RMSEA) ≤ 0.06. We derived reliability estimates from CFA using McDonald’s omega (ω) [34] and the estimator was weighted least squares with adjusted for mean and variances (WLSMV).

#### Network analysis

Networks consist of nodes (i.e., questionnaire items) connected through edges (associations) estimated using L1-regularized partial correlations (all nodes are regressed on each other adjusting for the effect of every other node). An L1-penalty is imposed on regression coefficients to balance goodness of fit and parsimony (also called the least absolute shrinkage and selection operator-*lasso*). Small edges are set to zero, which enables finding the sparsest (parsimonious) network and controls for multiple testing. As recommended, we used a tuning lambda = 0.25 [35]. We focused our analysis on expected influence node centrality, deemed more stable than other centrality measures [36]. We used multidimensional scaling for all graphs due to node distance interpretability (i.e., strongly associated nodes appear closer together, while weakly/negatively associated ones are more distant) [37]. We tested the accuracy of the networks using non-parametric bootstrapping procedures with *n* = 1000 runs. For centrality measures, we used a case-dropping bootstrap and evaluated the correlation coefficient of stability [CS (cor = 0.7)], which should be above 0.25, ideally above 0.5 [35]. Because PHQ-A and MFQ items may assess closely related constructs, we used the goldbricker procedure on each scale to check the data for node redundancy and possible item reduction [38]. Furthermore, to see if MFQ DSM and non-DSM items would cluster together or independently, we used the walktrap algorithm [39] to detect item clusters. Lastly, we used the network comparison test (NCT) [40] to compare PHQ-A and MFQ networks (the M statistic) according to sex. The same analysis was done for examining PHQ-A items and DSM items derived from the MFQ. Analyses were conducted in *R*, version 3.6.1 [41]. The R code is available in the Online Resources Material.

## Results

### Descriptive statistics

The PHQ-A sample included 7,720 participants (54.9% females), with a median PHQ-A total score of 8 (IQR = 10; Table S1). Over half (59.9%) of participants self-reported as white (Table S1). Females had higher median PHQ-A total scores than males (11 and 6, respectively; Mann–Whitney *U*-statistic = 446, *p* < 0.001). The most commonly endorsed items in the “nearly every day” option were sleep problems (27.3%), fatigue (23.5%) and feelings of worthlessness (23.4%). The average correlation between items was *r* = 0.39 (range *r* = 0.31 to *r* = 0.62; Online Resources Figure S1). There was negligible evidence of school-clustering (ICC = 0.009, 95% CI 0.004–0.017).

The MFQ sample included 1,070 participants (55.5% females), with a median MFQ total score of 19 (IQR = 20; Table S1). Females had higher median MFQ total scores than males (25 and 14, respectively; Mann–Whitney *U*-statistic = 744, *p* < 0.001). The most commonly endorsed items in the “always true” category were “It was hard to make decisions” (32.9%), followed by “I felt lonely” (26.1%) and “I felt sulky or upset with my parents” (24.7%). The average correlation between items was *r* = 0.31 (range *r* = − 0.25 to *r* = 0.69; see Figure S2 for a correlation matrix). DSM and non-DSM features were not different regarding medians (Mann–Whitney *U*-statistic = 133, *p* = 0.999) or standard deviations (Mann–Whitney *U*-statistic = 121, *p* = 0.615), suggesting neither group was more severe or variable than the other. There was a close to zero effect of school-clustering (ICC = − 0.004, 95% CI − 0.005 to 0.006).

### Confirmatory factor analysis for the PHQ-A

The unidimensional solution for the PHQ-A had good fit indices (CFI = 0.982, TLI = 0.976, RMSEA = 0.064) with adequate reliability (*ω* = 0.854, 95% CI 0.849–0.859). Items assessing suicidality had the highest initial thresholds (i.e., required higher depression severity to endorse the response option “Several days” over “None”), followed by psychomotor changes and concentration difficulties (Online Resources Table S4).

### PHQ-A network analysis

**Fig. 1:**
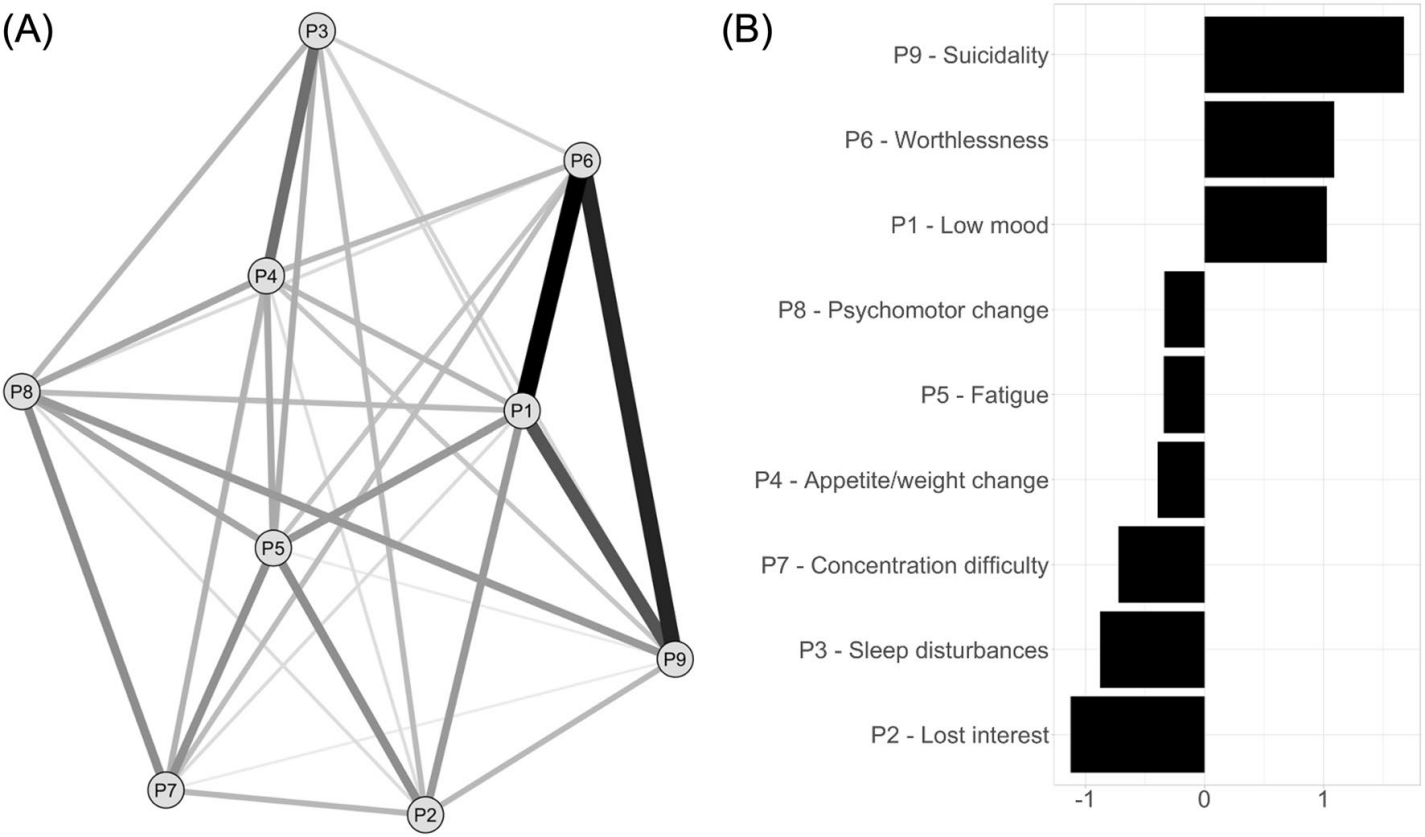
Network structure (**A**) and expected influence centrality (**B**) for the PHQ-A sample (*n* = 7720). *PHQ-A* Patient Health Questionnaire—Adolescent Version. **A** The lines represent positive associations. Line thickness and saturation represent correlation magnitude. The graph’s layout is based on multidimensional scaling, meaning closely associated nodes are placed closer together. **B**, The Y-axis shows PHQ items ordered from highest to lowest expected influence centrality; on the *X*-axis are *z*-standardized expected influence centrality values with zero as the mean value

Figure 1 presents the PHQ-A network structure. There were 35 non-zero edges out of 36 possible edges, with a mean weight of 0.10. There were strong partial correlations between low mood, feelings of worthlessness and suicidality items. Suicidality, low mood and feelings of worthlessness had the highest expected centrality indices (Fig. 1b). There was no suggestion of node redundancy from the goldbricker procedure. Males and females did not have different network structures (*M* = 0.068, *p* = 0.126), but there was a significant difference in overall connectivity, with females showing higher values than males (*S* = 0.201, *p* < 0.001; Online Resources Figure S3).

### MFQ confirmatory factor analysis

The unidimensional solution for the MFQ had adequate fit indices (CFI = 0.953, TLI = 0.949, RMSEA = 0.057) with good reliability estimates (*ω* = 0.941, 95% CI 0.936–0.946). Items assessing concentration difficulties had the lowest initial thresholds, while items reflecting psychomotor retardation (“I spoke slower than usual”) and suicidality (“I thought about killing myself”) had the highest initial thresholds (Online Resources Table S5).

### MFQ network analysis

**Fig. 2:**
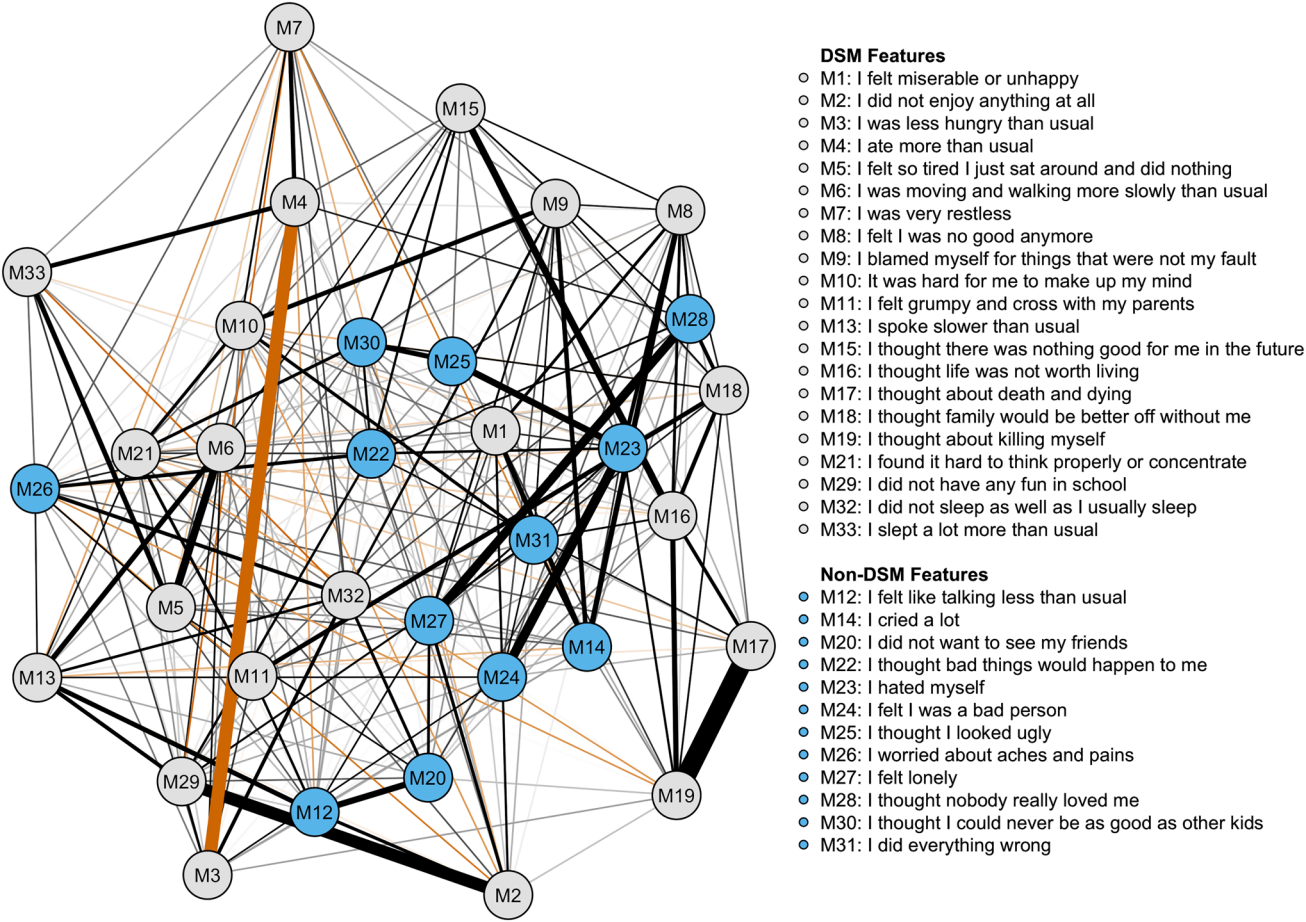
Network structure for the MFQ (*n* = 1070). *MFQ* Mood and Feelings Questionnaire. Gray nodes are symptoms included in the Diagnostic and Statistical Manual of Mental Disorders (5th edition) criteria for major depressive disorder, while blue nodes are symptoms not included in it. Black lines represent positive associations, while orange lines represent negative associations. Line thickness and saturation represent correlation magnitude. The layout is based on multidimensional scaling, meaning closely associated nodes are placed closer together

**Fig. 3:**
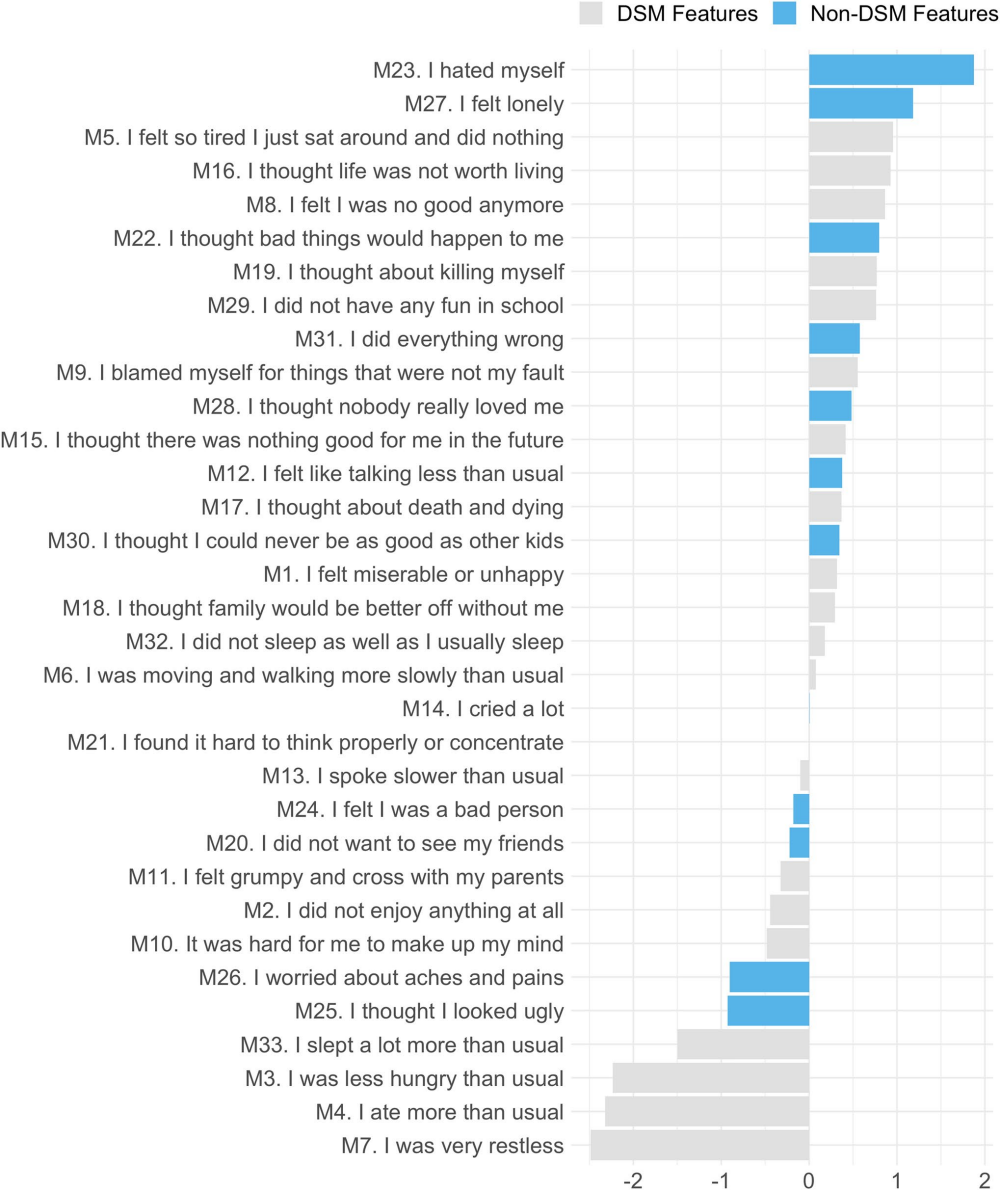
Expected influence centrality derived from the MFQ network (*n* = 1070). Gray bars represent items included in the Diagnostic and Statistical Manual of Mental Disorders (5th edition) major depressive disorder criteria, while blue bars represent items not included in the DSM. On the *Y*-axis, MFQ items are ordered by highest to lowest expected influence centrality; on the *X*-axis are the *z*-standardized expected influence centrality values with zero as the mean value

Figure 2 presents the MFQ sample network structure. There were 271 non-zero edges out of 528 possible edges, with a mean weight of 0.02. In contrast to the PHQ-A sample, low mood was not among the most central items. Rather, “hated myself”, “I felt lonely” and “I did not sleep as well as I usually sleep” were the most central items (Fig. 3). However, two of the three least central items were also non-DSM criteria (“I worried about aches and pains” and “I did not want to see my friends”). DSM and non-DSM items did not differ regarding their mean centrality (*W* = 151, *p* = 0.529), suggesting groups were not differentiated based on expected influence. The *walktrap* algorithm did not suggest DSM and non-DSM items to cluster independently—rather, as a complex, highly interconnected network of symptoms. Analyzing the network structure using an “or” rule to estimate DSM criteria from the MFQ items, the most central items were the same as in the full scale analysis in Figs. 2 and 3. Additionally, in a DSM-only MFQ analysis using an “or” rule, worthlessness, low mood and suicidality were the most central items (Online Resources Table S2 and Figures S4-S6). This is consistent with results from the PHQ-A analysis.

Males and females had different network structures (*M* = 0.272, *p* < 0.001), with no difference in overall connectivity (*S* = 0.400, *p* = 0.379). “I hated myself” was the most central items for boys and girls, followed by “I felt lonely” and “I thought bad things would happen to me” for males and “I felt I was no good anymore” and “I thought life was not worth living” for females (Online Resources Figure S7). Items M2 (“I did not enjoy anything at all”), M14 (“I cried a lot”) and M17 (“I thought about death and dying”) were more central for females, while items M4 (“I ate more than usual”) and M33 (“I slept a lot more than usual”) were more central for males. Examining only MFQ DSM items, there was no significant differences in network structure (*M* = 0.122, *p* = 0.73) or connectivity (*S* = 0.084, *p* = 0.33) for either sex.

### Network accuracy

The PHQ-A and the MFQ showed adequate network structure accuracy, with non-zero weights in bootstrapped difference tests (*α* = 0.05). For both scales, most edges were significantly different. Centrality estimates for both the PHQ-A and the MFQ expected influence had optimal levels of stability (CS-coefficient > 0.75) and were not biased by node variance (PHQ-A: *r* = − 0.111, *p* = 0.777. MFQ: *r* = − 0.042, *p* = 0.814). All graphs are available upon request.

## Discussion

In two similarly recruited independent school-based samples from Brazil, we examined, using latent and network analyses, the characteristics of adolescent depression features that are and are not included in the formal DSM criteria for MDD. In the PHQ-A sample—including exclusively DSM items—we found low mood and feelings of worthlessness as the two most central items. In the MFQ sample, we found DSM items to be part of a complex and interconnected network that also includes items not explicitly captured by the DSM criteria for MDD. In this sample, the two most central features were self-hatred and loneliness – features not overtly captured by the DSM.

Depression is widely acknowledged as a heterogeneous construct [7, 12]. We attempted to tackle such heterogeneity by examining two-dimensional measures of depressive symptoms: the PHQ-A, a widely used instrument reflecting strict DSM adolescent MDD criteria; and the MFQ, which includes those criteria as well as features not included in the DSM. The PHQ-9, from which the PHQ-A is derived and closely related to, is one of the standardized mental health outcomes recently proposed by the Wellcome Trust and the National Institute of Mental Health as an attempt to harmonize data from different research settings [42]. Meanwhile, the MFQ was used as the main outcome for the largest clinical trial of psychotherapy in adolescents with depression [43].

Our work is in agreement with previous findings from high-income countries showing self-hatred and loneliness as among the most interconnected items in community-based samples of adolescents [17, 36]. Our results are also in line with a previous report of middle- and high-school students in the United States that found self-hatred, loneliness, sadness and worthlessness as the most central symptoms of adolescent depression using the short version of the MFQ [18]. Moreover, our work replicates and expands on findings from two studies that show non-DSM features to be as important in depression networks as DSM criteria [23, 24]—results derived from adult clinical samples. Our report adds to these studies by applying both latent and network approaches to two non-clinical, school-based adolescent samples from a middle-income country.

An important implication of our findings is the question of whether the DSM, through its consensus-based operationalization of adolescent MDD, is capturing all features of depression that are important to the young people experiencing this disorder. In the PHQ-A sample, excessive guilt and/or feelings of worthlessness was a highly central item, while anhedonia, one of the cardinal symptoms of MDD, was not. In the MFQ sample, self-hatred and low self-esteem were highly central nodes, though neither is explicitly and adequately captured by the DSM’s criteria A7 of “feelings of worthlessness and/or excessive guilt”. Both are, however, predictors and/or markers of negative outcomes longitudinally associated with depression [44, 45]. The same holds true for loneliness, also found to be highly central in our report and not mentioned as one of the nine MDD DSM criteria [44]. A recent qualitative meta-synthesis also identified loneliness as a central experience among young people with depression [46]. Moreover, findings from developmental social neuroscience research suggest that adolescence is a period of increased vulnerability to perceived loneliness, and loneliness is associated with heightened adverse responses to social cues in functional neuroimaging studies [47].

Interestingly, three of the five most central items in the MFQ network (“I hated myself”, “I thought life was not worth living”, “I thought bad things would happen to me”) parallel Beck’s cognitive triad of negative views about the self, the world and the future [48]. Furthermore, hopelessness, considered by 11th version of the International Classification of Diseases (ICD-11) as an accessory symptom of depression [49] and shown to be highly central in our adolescent sample, was shown to better differentiate depressed and non-depressed adults according to DSM-IV criteria [50]. Our results come at a time of growing interest in understanding outcomes based on patients’ needs and priorities. In accordance with our results, Chevance and colleagues found, among other domains, improvements in feelings of loneliness, low self-esteem and social isolation to be commonly cited expected benefits of depression treatment [11]. A systematic review of qualitative studies of adults showed only 7 out of 15 frequently mentioned features of depression from worldwide samples are part of the DSM criteria for MDD diagnosis, with loneliness notably being the fourth most frequently mentioned symptom among Western and non-Western populations [51]. Symptoms tended to have significant variability across cultures, suggesting DSM criteria may also miss important information in culturally diverse settings.

Although useful for clinical and research purposes, there has been growing skepticism regarding the adequacy of the consensus-based approach to psychopathology used by the DSM [15]. Different conceptualizations of depression, with empirical decisions to add or drop symptoms, are common within the history of psychiatry [15]. It is possible that, given the biopsychosocial particularities of adolescence as a life period, simply extending the definition of MDD for adulthood to adolescence, with the inclusion of irritability as an alternative to depressed mood in the A1 criterion [6], may not fully encompass particular characteristics of how young people experience depressive symptomatology. Highly central nodes in our results such as pessimism and hopelessness are important clinical features of depression [11, 12, 48], but neither is adequately captured by the DSM A7 criteria of excessive guilt and worthlessness [12]. Importantly, a recent study of depressed parents and their offspring did not support irritability as being more common in adolescents than in adults, though it did find different symptomatic profiles according to age [52]. Indeed, irritability has been suggested as an antecedent of low mood in longitudinal research and/or as a marker of severity [53]. In the PHQ-A sample, the item questioning low mood or irritability was highly central—though, following DSM criteria, there was a single item simultaneously questioning both symptoms. In the MFQ sample, irritability was not a specially interconnected node.

The past decade has seen the rise of data-driven methods for more refined understanding of depressive phenotypes. We used network analysis as an exploratory approach for studying relations among depressive symptoms in adolescence. Other data-driven approaches have been used to better understand symptom clusters of treatment response in adolescents [54]. However, a systematic review exposed difficulties in finding data-driven subtypes that may stem from the over-reliance on DSM criteria as well as on the common cause model [55]. As an alternative to these shortcomings, the network approach advances psychopathological research by considering symptoms as mutually reinforcing entities [16]. By combining individuals with very different symptom profiles into an unweighted sum-score, we risk losing important connections that are fundamental to continue progress in depression research [16]. Interestingly, results from our MFQ sample did not support a clear separation of DSM and non-DSM criteria. Using regularized partial correlations, which calculate symptom-symptom correlations adjusting for every other symptom in the network, we found all items to be part of a highly interconnected network. Though increasing the number of symptoms contemplated by the DSM certainly could increase MDD’s heterogeneity, not properly evaluating important non-DSM features also hinders understanding of how young people experience the disorder. Although most of the network literature to date has used cross-sectional data, these can be useful for exploring singular patterns of symptom association as a data-driven, hypothesis-generating approach. Further studies assessing longitudinal datasets will be crucial to better understand the developmental presentation of depressive symptomatology in adolescence.

Even though we are considering MFQ non-DSM items as part of the depression spectrum, it is conceivable that non-DSM MFQ items may capture a different construct, not necessarily depression, but related to a comorbid mental disorder. It is plausible that the MFQ, even if a priori designed to encompass symptoms of depression, actually captures anxiety symptoms or broader psychopathological distress. As much as our findings suggest a potential expansion of the depressive syndrome, stakeholders may share different propositions on an even larger expansion, not exclusively or fully captured by psychopathology research using more traditional measurements [11]. This is important in a larger discussion on the distinction between what are the disorder’s diagnostic criteria and the disorder itself. Our argument of a potential insufficiency of DSM criteria for adolescent MDD is in line with an indexical view of nosology [13]—symptoms suggest the presence of the disorder, but they are not fully explanatory of it. Rather, these are possible alterations reflective of the condition. If we consider that the diagnostic criteria (i.e., DSM criteria for adolescent MDD) are the only means of identifying depression, we may miss more detailed information of the range of depressive experiences in teenagers (i.e., features included and not included in the DSM criteria). These concerns have been previously raised in network examinations of adult samples [23, 24] and are even more pertinent in studies of adolescent features of depression. Despite the DSM’s numerous contributions and for allowing multiple advances in psychopathology research, interpreting the diagnostic criteria as full descriptions of the syndrome of depression among adolescents may be insufficient for understanding its uniqueness and peculiarities. Acknowledging limitations of psychopathology research [11] and a possible overlook on what is most important to patients is crucial for advancing depression research.

A number of limitations must be noted. Firstly, our results are based on cross-sectional data from school-based samples, which simultaneously precludes necessary generalization of findings to other populations or clinical samples and highlights the need for longitudinal research for further disentangling of results as specific features of adolescent depression. Although both samples were recruited using closely related protocols, respondents were different participants, which impedes direct comparisons between scales, as well as possible risk factor exposition (see Table S1). Also, it is worth noting the high frequency of endorsement of the seven questions on maltreatment (see reference [28] for details) in both samples—which is a limitation in terms of external generalizability but also emphasizes importance of studying adolescent depression in socially vulnerable populations (i.e., public state schools in a middle-income country). Furthermore, comparisons between the PHQ-A and DSM items derived from the MFQ were drawn from an “or” rule based on face validity and item content, suggesting caution in evaluating these results. It is worth mentioning that there were significant differences in network structure between the two scales, but not of centrality estimates. This may be due to a potential impact of the number of questionnaire items on response pattern of a 9-item and a 33-item questionnaire, the “or” rule used to derive DSM features from the MFQ and the different samples. Consequently, the replicability and longitudinal dynamics of network characteristics, influence of context and number of items in network estimation are matters of continued interest that deserve further investigation [56, 57]. Additionally, the PHQ-9, from which the PHQ-A is derived, has been under heavy criticism for its accuracy and psychometric properties [58]. Both the PHQ-A and the MFQ, as self-report instruments, may lead to biases in terms of symptom reporting when compared to clinician-rated scales or structured interviews [59]. Because this report is based on data from the screening phase of a larger [28], clinical diagnosis was not possible for either sample. We are not aware of any other study examining depressive symptoms with the PHQ-A or the MFQ in Brazilian adolescents, thus limiting comparisons between universal and local symptom conceptualizations. Finally, we used only one instrument to examine the centrality of DSM and non-DSM criteria, which could have biased the findings.

In light of these limitations, and considering we are at the early stages of implementing network techniques to adolescent psychopathology, we believe our study had several strengths. The use of two large, community-based samples allows for the study of depression symptom presentation in a setting that is closer to the real-world and, therefore, may enhance our comprehension of the dimensional presentation of depression in adolescence. Furthermore, by recruiting adolescents in the school environment, we avoid a severity bias from clinical referrals and selection bias in contexts of scarcer resources. Additionally, the somewhat narrow age range of participants (14–16 years-old), despite limiting to some extent immediate extrapolations to younger or older individuals, increases sample homogeneity. Furthermore, the analysis of two different scales with two different but complementary analytical approaches allows for an in-depth examination of MDD’s heterogeneity in outcome measures [60], as well as an investigation of the relations of DSM and non-DSM items. Even though DSM and non-DSM criteria tend to be related, the use of regularized partial correlations allow for multiple comparison adjustment and finding the most parsimonious network structure and centrality estimates. By combining MFQ items to more closely resemble DSM criteria using the “or” rule, we were better suited to distinguish between DSM and non-DSM criteria and allowed some comparability between the PHQ-A and the MFQ scales. Additionally, applying data-driven symptom-level techniques acknowledges growing support for the study of particular symptoms instead of unweighted sum-scores [16]. Nevertheless, we should mention the importance of replicating our findings in other settings (e.g., more resource-deprived countries), in other populations (e.g., in- and out-patient depressed adolescents or community-based youths) and with longitudinal study designs.

In summary, the present report aimed to examine the dimensional structure of two commonly used depression scales in two similarly recruited independent adolescent samples in a middle-income setting. Our study expands on previous literature in adult samples showing DSM and non-DSM features to be part of an interconnected network of symptoms [23, 24]. Our findings suggest DSM criteria for MDD not to be more frequent, more severe or more interconnected than non-DSM items, but instead both appear to be part of a larger network of adolescent depression symptoms. Refining our insights into clinical presentation of depressive symptoms in adolescence may have significant clinical implications for our understanding of such a burdensome condition for young people.

## Supplementary Information

Below is the link to the electronic supplementary material.

Supplementary file1 (DOCX 5973 KB)

## Data Availability

Data are available upon reasonable request.

## Code availability

All R code for analysis on the main text and on the Online Resources are available at the Open Science Framework https://osf.io/uvbh3/
