# Supplementary material for: Adolescent depression beyond DSM definition: a network analysis
Source: Eur Child Adolesc Psychiatry. 2021 Dec 2;32(5):881–92. doi: 10.1007/s00787-021-01908-1 (PMC10147766; doi:10.1007/s00787-021-01908-1)
Supplement: Supplementary file 1 — Supplementary file1 (DOCX 5973 KB) [file 787_2021_1908_MOESM1_ESM.docx]

Supplementary materials for **Adolescent depression beyond DSM definition: a network analysis**

Pedro H. Manfro, Rivka B. Pereira, Martha Rosa, Hugo Cogo-Moreira, Helen L. Fisher, Brandon A. Kohrt, Valeria Mondelli, Christian Kieling

All R code for analysis on the main text and on the supplementary material are available at the Open Science Framework https://osf.io/uvbh3/

Table of Contents

[Supplementary Table S1. Descriptive statistics for the PHQ-A (n=7,720) and the MFQ (n=1,070) samples 3](#_Toc75810881)

[Supplementary Table S2. DSM, PHQ-A and MFQ item comparison 3](#_Toc75810882)

[Supplementary Table S4. Summary statistics for the PHQ-A and MFQ items 7](#_Toc75810883)

[Supplementary Figure S1. PHQ-A items correlation matrix 9](#_Toc75810884)

[Supplementary Figure S2: Correlation matrix from the MFQ items 10](#_Toc75810885)

[Supplementary Table S5. Confirmatory factor analysis (CFA) factor loadings, reliability and fit indices for the PHQ-A 11](#_Toc75810886)

[Supplementary Figure S3. Expected influence centrality of males and females from the PHQ-A network structure 12](#_Toc75810887)

[Supplementary Table S6. CFA factor loadings, reliability and fit indices for the MFQ 13](#_Toc75810888)

[Supplementary Figure S4. Network structure for the MFQ (n=1,070) with DSM and non-DSM symptoms 15](#_Toc75810889)

[Supplementary Figure S5: Expected influence centrality for the MFQ (n=1,070) with DSM and non-DSM symptoms 16](#_Toc75810890)

[Supplementary Figure S6. Network structure of PHQ-A items (A) and DSM items derived from the MFQ with an “or” rule (B; see Table S1 for a full explanation) 17](#_Toc75810891)

[Supplementary Figure S7. Expected influence centrality of PHQ-A items and DSM items derived from the MFQ with an “or” rule 19](#_Toc75810892)

[Supplementary Figure S8. Expected influence centrality of males (blue) and females (red) from the MFQ network 20](#_Toc75810893)

[REFERENCES 22](#_Toc75810894)

| Supplementary Table S1. Descriptive statistics for the PHQ-A (n=7,720) and the MFQ (n=1,070) samples | | | | |
| --- | --- | --- | --- | --- |
|  | **Non-imputed**  **PHQ-A sample (n=7,288)** | **PHQ-A Sample (n=7,720)** | **Non-imputed**  **MFQ sample (n=944)** | **MFQ Sample (n=1,070)** |
| Females (%, 95%CI) | 4,022 (55.2, 54.0-56.3%) | 4,241 (54.9%, 53.8-56.0%) | 518 (54.9%, 51.6-58.1%) | 594 (55.5%, 52.5-58.4%) |
| Mean Age (SD, 95%CI) | 15.73 (0.52, 15.70-15.76) | 15.74 (0.8, 15.72-15.75) | 15.73(0.76, 15.70-15.76) | 15.75 (0.74, 15.70-15.79) |
| Childhood maltreatment (%, 95%CI) |  |  |  |  |
| None | 2047 (28.1%, 27.1-29.1%) | 2157 (27.9%, 26.9-28.9%) | 432 (45.8%, 42.5-49.0%) | 487 (45.5%, 42.5-48.5%) |
| Probable | 1643 (22.5%, 21.6-23.5%) | 1743 (22.5%, 21.6-23.5%) | 222 (23.5%, 20.8-26.4%) | 243 (22.7%, 20.3-25.3%) |
| Severe | 3728 (51.1%, 50.0-52.2%) | 3820 (49.4%, 48.3-50.5%) | 290 (30.7%, 27.8-33.7%) | 340 (31.7%, 29.0-34.6%) |
| Median PHQ-A (SD, 95%CI) | 8 (6.53, 9.00-9.50) | 8(6.52, 9.00-9.53) | - | - |
| Range PHQ-A (IQR) | 0-27 (4-14) | 0-27 (4-14) | - | - |
| Skewness PHQ-A sum-score (kurtosis) | 0.62(-0.47) | 0.63 (-0.46) | - | - |
| Median MFQ (SD, 95%CI) | - | - | 19 (13.62, 19.99-21.99) | 17(13.92, 20.00-20.92) |
| Range MFQ (IQR) | - | - | 0-66 (11-31) | 0-66 (11-31) |
| Skewness MFQ sum-score (kurtosis) | - | - | -0.67(0.44) | 0.68 (-0.45) |
| Skin color (white, %, 95%CI) | 4,399 (60.4%, 59.2-61.5%) | 4,630 (59.9%, 58.8-61.0%) | 563 (59.6, 56.4-62.8%) | 635 (59.3%, 56.3-62.2%) |
| Note. PHQ-A: Patient Health Questionnaire – Adolescent Version. MFQ: Mood and Feelings Questionnaire. 95%CI: 95% confidence interval. SD: standard deviation. The maltreatment variable is divided into “none” (no positive answer for items on emotional abuse, emotional neglect, physical abuse, physical neglect or sexual abuse), "probable” (one positive answer) and "severe" (two or more positive answer) as per previous literature[1,2]. The skin color item followed Brazilian official census (IBGE) of self-reported categories (white/yellow/indigenous/brown/black). For analyses, two categories (white vs. non-white) were formed. IQR: Interquartile range | | | | |

| Supplementary Table S2. DSM, PHQ-A and MFQ item comparison | | |
| --- | --- | --- |
| DSM items | **PHQ-A items** | **MFQ items** |
| A1. Depressed mood most of the day, nearly every day, as indicated by either subjective report (e.g., feels sad, empty, hopeless) or observation made by others (e.g., appears tearful). (Note: In children and adolescents, can be irritable mood.) | P1. Feeling down, depressed, irritable, or hopeless? | M1. I felt miserable or unhappy.  M11. I felt grumpy and cross with my parents.  M14. I cried a lot.  M15. I thought there was nothing good for me in the future. |
| A2. Markedly diminished interest or pleasure in all, or almost all, activities most of the day, nearly every day (as indicated by either subjective account or observation). | P2. Little interest or pleasure in doing things? | M2. I didn’t enjoy anything at all.  M29. I didn’t have any fun in school. |
| A3. Significant weight loss when not dieting or weight gain (e.g., a change of more than 5% of body weight in a month), or decrease or increase in appetite nearly every day. (Note: In children, consider failure to make expected weight gain.) | P4. Poor appetite, weight loss, or overeating? | M3. I was less hungry than usual.  M4. I ate more than usual. |
| A4. Insomnia or hypersomnia nearly every day. | P3. Trouble falling asleep, staying asleep, or sleeping too much? | M32. I didn’t sleep as well as I usually sleep.  M33. I slept a lot more than usual. |
| A5. Psychomotor agitation or retardation nearly every day (observable by others, not merely subjective feelings of restlessness or being slowed down). | P8. Moving or speaking so slowly that other people could have noticed? Or the opposite – being so fidgety or restless that you were moving around a lot more than usual? | M6. I was moving and walking more slowly than usual.  M7. I was very restless.  M13. I was talking more slowly than usual. |
| A6. Fatigue or loss of energy nearly every day. | P5. Feeling tired, or having little energy? | M5. I felt so tired I just sat around and did nothing. |
| A7. Feelings of worthlessness or excessive or inappropriate guilt (which may be delusional) nearly every day (not merely self-reproach or guilt about being sick). | P6. Feeling bad about yourself – or feeling that you are a failure, or that you have let yourself or your family down? | M8. I felt I was no good anymore.  M9. I blamed myself for things that weren’t my fault.  M24. I felt I was a bad person.  M25. I thought I looked ugly.  M28. I thought nobody really loved me.  M30. I thought I could never be as good as other kids.  M31. I did everything wrong. |
| A8. Diminished ability to think or concentrate, or indecisiveness, nearly every day (either by subjective account or as observed by others). | P7. Trouble concentrating on things like school work, reading, or watching TV? | M10. It was hard for me to make up my mind.  M21. I found it hard to think properly or concentrate. |
| A9. Recurrent thoughts of death (not just fear of dying), recurrent suicidal ideation without a specific plan, or a suicide attempt or a specific plan for committing suicide | P9. Thoughts that you would be better off dead, or of hurting yourself in some way? | M16. I thought that life wasn’t worth living.  M17. I thought about death or dying.  M18. I thought my family would be better off without me.  M19. I thought about killing myself. |
| PHQ-A: Patient Health Questionnaire – Adolescent Version. MFQ: Mood and Feelings Questionnaire. MFQ items range: 0 (not true), 1 (sometimes true), 2 (true). For comparison between PHQ-A and MFQ items, we used an “or” rule to estimate 9 DSM criteria from the 33 MFQ items – e.g., if any one of items M16 *or* M17 *or* M18 *or* M19 were endorsed as 2 (true), we considered it equivalent to endorsing the A9 criteria of suicidality as 2 (true); if three of the four aforementioned items were endorsed as 1 (sometimes true) and one of them was considered as 2 (true), we also considered it equivalent to endorsing the A9 criteria as 2 (true); if any three of the four items were endorsed as 0 (not true) and one was endorsed as 1 (sometimes true), we considered it equivalent to endorsing the A9 criteria as 1 (sometimes true). | | |
|  | | |

| **Supplementary Table S3.** Non-DSM MFQ items |
| --- |
| MFQ12. I felt like talking less than usual. |
| MFQ14. I cried a lot. |
| MFQ15. I thought there was nothing good for me in the future. |
| MFQ20. I didn’t want to see my friends. |
| MFQ22. I thought bad things would happen to me. |
| MFQ23. I hated myself. |
| MFQ24. I felt I was a bad person. |
| MFQ25. I thought I looked ugly. |
| MFQ26. I worried about aches and pains. |
| MFQ27. I felt lonely. |
| MFQ28. I thought nobody really loved me. |
| MFQ30. I thought I could never be as good as other kids. |
| MFQ31. I did everything wrong. |
| MFQ: Mood and Feelings Questionnaire. We classified MFQ items as “non-DSM” according to previous studies [3–5]. |

| Supplementary Table S4. Summary statistics for the PHQ-A and MFQ items | | | | | |
| --- | --- | --- | --- | --- | --- |
| **PHQ-A items** | **Mean** | **SD** | **MFQ items** | **Mean** | **SD** |
| P1 - Low mood | 1.25 | 1.06 | M1 - I felt miserable or unhappy | 0.74 | 0.67 |
| P2 - Lost interest | 1.23 | 1.01 | M2 - I did not enjoy anything at all | 0.29 | 0.52 |
| P3 - Sleep disturbances | 1.32 | 1.19 | M3 - I was less hungry than usual | 0.51 | 0.73 |
| P4 - Appetite/weight change | 1.02 | 1.13 | M4 - I ate more than usual | 0.77 | 0.75 |
| P5 - Fatigue | 1.39 | 1.07 | M5 - I felt so tired I just sat around and did nothing | 0.89 | 0.79 |
| P6 - Worthlessness | 1.18 | 1.17 | M6 - I was moving and walking more slowly than usual | 0.48 | 0.70 |
| P7 - Concentration difficulty | 0.79 | 1.03 | M7 - I was very restless | 0.82 | 0.73 |
| P8 - Psychomotor change | 0.73 | 0.99 | M8 - I felt I was no good anymore | 0.51 | 0.73 |
| P9 - Suicidality | 0.57 | 0.96 | M9 - I blamed myself for things that were not my fault | 0.70 | 0.79 |
|  |  |  | M10 - It was hard for me to make up my mind | 1.08 | 0.75 |
|  |  |  | M11 - I felt grumpy and cross with my parents | 0.82 | 0.80 |
|  |  |  | M12 - I felt like talking less than usual | 0.74 | 0.79 |
|  |  |  | M13 - I spoke slower than usual | 0.24 | 0.53 |
|  |  |  | M14 - I cried a lot | 0.53 | 0.73 |
|  |  |  | M15 - I thought there was nothing good for me in the future | 0.61 | 0.76 |
|  |  |  | M16 - I thought life was not worth living | 0.39 | 0.68 |
|  |  |  | M17 - I thought about death and dying | 0.51 | 0.74 |
|  |  |  | M18 - I thought family would be better off without me | 0.42 | 0.69 |
|  |  |  | M19 - I thought about killing myself | 0.28 | 0.61 |
|  |  |  | M20 - I did not want to see my friends | 0.34 | 0.60 |
|  |  |  | M21 - I found it hard to think properly or concentrate | 0.89 | 0.72 |
|  |  |  | M22 - I thought bad things would happen to me | 0.70 | 0.76 |
|  |  |  | M23 - I hated myself | 0.53 | 0.75 |
|  |  |  | M24 - I felt I was a bad person | 0.51 | 0.72 |
|  |  |  | M25 - I thought I looked ugly | 0.81 | 0.80 |
|  |  |  | M26 - I worried about aches and pains | 0.66 | 0.77 |
|  |  |  | M27 - I felt lonely | 0.82 | 0.81 |
|  |  |  | M28 - I thought nobody really loved me | 0.52 | 0.75 |
|  |  |  | M29 - I did not have any fun in school | 0.34 | 0.57 |
|  |  |  | M30 - I thought I could never be as good as other kids | 0.66 | 0.79 |
|  |  |  | M31 - I did everything wrong | 0.52 | 0.70 |
|  |  |  | M32 - I did not sleep as well as I usually sleep | 0.76 | 0.81 |
|  |  |  | M33 - I slept a lot more than usual | 0.75 | 0.80 |
| PHQ-A: Patient Health Questionnaire – Adolescent Version. MFQ: Mood and Feelings Questionnaire. SD: standard deviation. PHQ-A range: 0 (none), 1 (several days), 2 (more than half the days) and 3 (nearly every day). MFQ items range: 0 (not true), 1 (sometimes true), 2 (true). Of note, the PHQ-A has 4 response options, while the MFQ has 3; therefore, PHQ-A items will have higher means and standard deviations than MFQ’s. | | | | | |

# Supplementary Figure S1. Spearman correlation matrix from the PHQ-A items


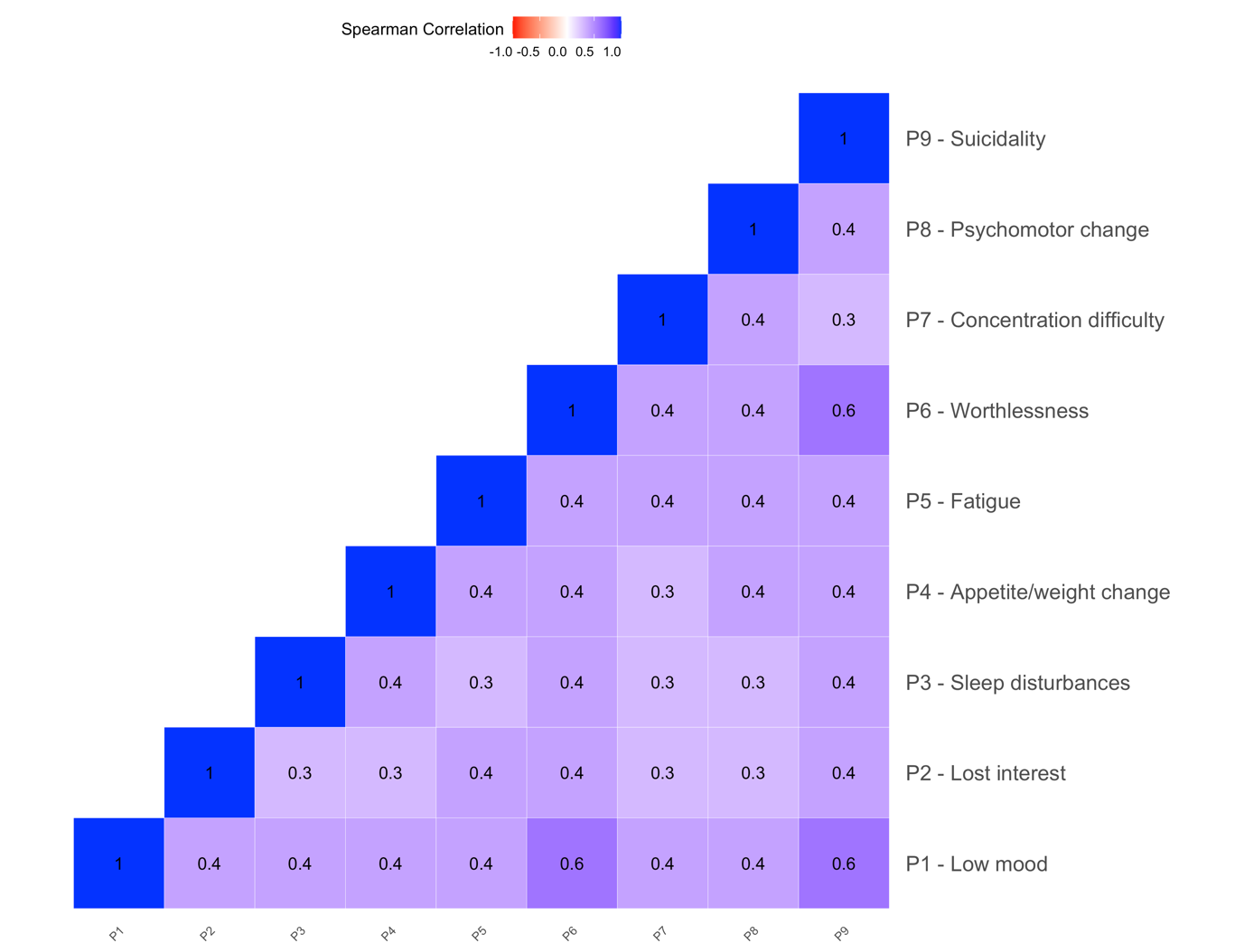


Note. PHQ-A: Patient Health Questionnaire – Adolescent Version. The color gradient goes from blue (positive correlations) to red (negative correlations). Darker shades represent stronger correlations than lighter ones.

# Supplementary Figure S2: Spearman correlation matrix from the MFQ items


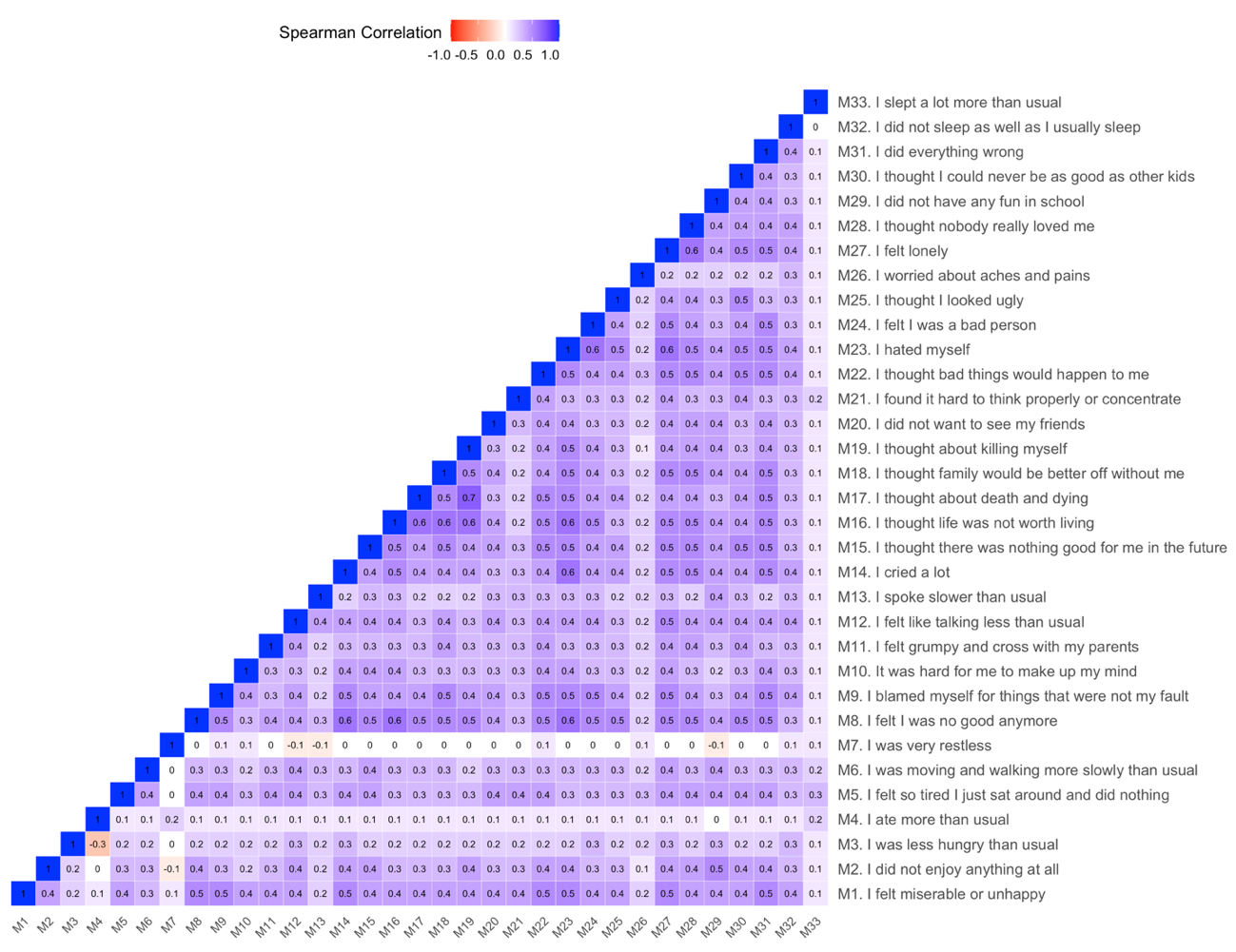


Note. MFQ: Mood and Feelings Questionnaire. As in Figure S1, the color gradient goes from blue (positive correlations) to red (negative correlations). Darker shades indicate stronger correlations.

| Supplementary Table S5. Confirmatory factor analysis (CFA) factor loadings, reliability and fit indices for the PHQ-A | | | | |
| --- | --- | --- | --- | --- |
| **PHQ-A items** | **Factor loadings (ƛ)** | **Thresholds (ƛ)** | | |
|  |  | 1 (several days) | 2 (more than half the days) | 3 (nearly every day) |
| P1 - Low mood | 0.821 | -0.604 | 0.443 | 0.867 |
| P2 - Lost interest | 0.616 | -0.630 | 0.417 | 1.015 |
| P3 - Sleep disturbances | 0.592 | -0.406 | 0.251 | 0.648 |
| P4 - Appetite/weight change | 0.647 | -0.104 | 0.507 | 0.958 |
| P5 - Fatigue | 0.650 | -0.747 | 0.279 | 0.761 |
| P6 - Worthlessness | 0.831 | -0.273 | 0.393 | 0.766 |
| P7 - Concentration difficulty | 0.603 | 0.095 | 0.767 | 1.198 |
| P8 - Psychomotor change | 0.654 | 0.164 | 0.848 | 1.281 |
| P9 - Suicidality | 0.846 | 0.452 | 1.016 | 1.302 |
| Note. PHQ-A: Patient Health Questionnaire – Adolescent Version. Factor loadings, fit indices and thresholds are presented for the unidimensional solution CFA results. Factor loadings, fit indices and thresholds are presented for the unidimensional solution CFA results. Fit indices for the unidimensional solution: McDonald’s Omega=0.854; CFI=0.982; TLI=0.976; RMSEA=0.064. Omega cut-off close to or above 0.7; CFI and TLI cut-offs close to or larger than 0.950; RMSEA cut-off close to or smaller than 0.060. Thresholds represent the necessary standardized latent value required to endorse a “harder” response option than an “easier” one (i.e., required higher depression severity in order to endorse the response option “Several days” over “None”). | | | | |

# Supplementary Figure S3. Expected influence centrality of males and females from the PHQ-A network structure


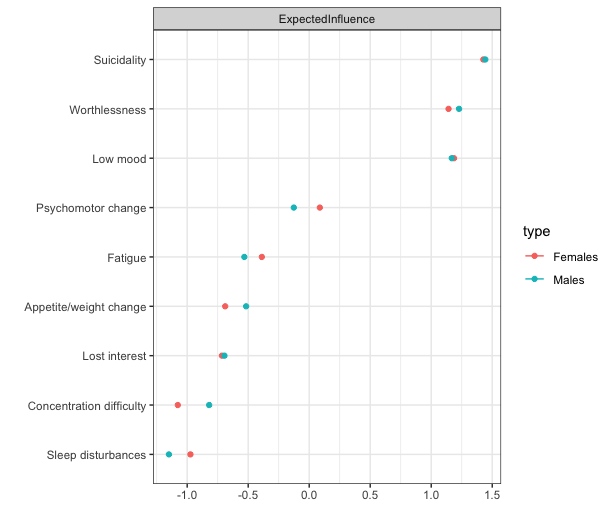


Note. PHQ-A: Patient Health Questionnaire – Adolescent Version. Blue points represent expected influence centrality derived from the PHQ-A network structure of males. Red points represent expected influence centrality derived from the PHQ-A network structure of females. Suicidality, worthlessness and low mood were the most central item for boys and girls. On the Y-Axis, PHQ-A items ordered by highest to lowest expected influence centrality; on the X-Axis are z-standardized expected influence centrality values with zero as the mean value.

| Supplementary Table S6. CFA factor loadings, reliability and fit indices for the MFQ | | | |
| --- | --- | --- | --- |
|  | **Factor loadings (ƛ)** | **Thresholds (ƛ)** | |
|  |  | 1 (Sometimes True) | 2 (True) |
| M1 - I felt miserable or unhappy | 0.767 | -0.292 | 1.131 |
| M2 - I did not enjoy anything at all | 0.686 | 0.655 | 1.817 |
| M3 - I was less hungry than usual | 0.436 | 0.337 | 1.063 |
| M4 - I ate more than usual | 0.172 | -0.193 | 0.848 |
| M5 - I felt so tired I just sat around and did nothing | 0.646 | -0.327 | 0.624 |
| M6 - I was moving and walking more slowly than usual | 0.589 | 0.351 | 1.158 |
| M7 - I was very restless | 0.029 | -0.332 | 0.865 |
| M8 - I felt I was no good anymore | 0.840 | 0.339 | 1.051 |
| M9 - I blamed myself for things that were not my fault | 0.756 | 0.023 | 0.799 |
| M10 - It was hard for me to make up my mind | 0.587 | -0.667 | 0.443 |
| M11 - I felt grumpy and cross with my parents | 0.603 | -0.189 | 0.685 |
| M12 - I felt like talking less than usual | 0.694 | -0.063 | 0.786 |
| M13 - I spoke slower than usual | 0.574 | 0.879 | 1.623 |
| M14 - I cried a lot | 0.777 | 0.278 | 1.047 |
| M15 - I thought there was nothing good for me in the future | 0.772 | 0.155 | 0.935 |
| M16 - I thought life was not worth living | 0.838 | 0.573 | 1.201 |
| M17 - I thought about death and dying | 0.810 | 0.354 | 1.019 |
| M18 - I thought family would be better off without me | 0.782 | 0.500 | 1.177 |
| M19 - I thought about killing myself | 0.850 | 0.848 | 1.372 |
| M20 - I did not want to see my friends | 0.683 | 0.613 | 1.455 |
| M21 - I found it hard to think properly or concentrate | 0.560 | -0.451 | 0.783 |
| M22 - I thought bad things would happen to me | 0.772 | -0.049 | 0.896 |
| M23 - I hated myself | 0.872 | 0.317 | 0.988 |
| M24 - I felt I was a bad person | 0.751 | 0.297 | 1.101 |
| M25 - I thought I looked ugly | 0.657 | -0.160 | 0.682 |
| M26 - I worried about aches and pains | 0.389 | 0.056 | 0.889 |
| M27 - I felt lonely | 0.823 | -0.155 | 0.641 |
| M28 - I thought nobody really loved me | 0.782 | 0.344 | 0.995 |
| M29 - I did not have any fun in school | 0.727 | 0.560 | 1.605 |
| M30 - I thought I could never be as good as other kids | 0.723 | 0.108 | 0.822 |
| M31 - I did everything wrong | 0.792 | 0.263 | 1.149 |
| M32 - I did not sleep as well as I usually sleep | 0.603 | -0.052 | 0.709 |
| M33 - I slept a lot more than usual | 0.227 | -0.054 | 0.724 |
| Note. PHQ-A: Patient Health Questionnaire – Adolescent Version. MFQ: Mood and Feelings Questionnaire. Factor loadings, fit indices and thresholds are presented for the unidimensional solution CFA results. Fit indices for the unidimensional solution: McDonald’s Omega=0.941; CFI=0.951; TLI=0.948; RMSEA=0.058. Omega cut-off close to or above 0.7; CFI and TLI cut-offs close to or larger than 0.950; RMSEA cut-off close to or smaller than 0.060. Thresholds represent the necessary standardized latent value required to endorse a “harder” response option than an “easier” one (i.e., required higher depression severity in order to endorse the response option “Sometimes true” over “Not true”). | | | |

# Supplementary Figure S4. Network structure for the MFQ (n=1,070) with DSM and non-DSM features

**
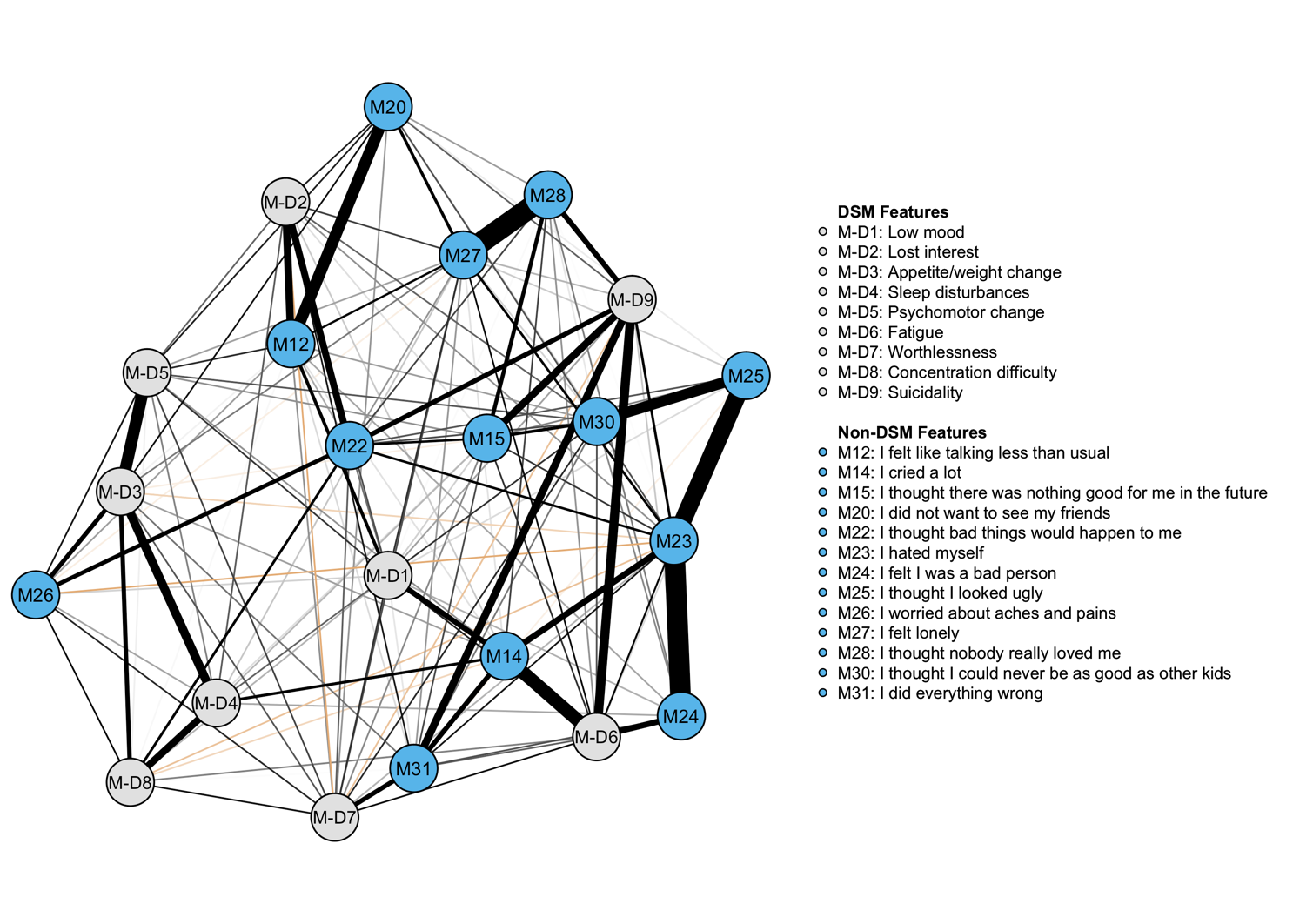
**

Note. MFQ: Mood and Feelings Questionnaire. Gray nodes are the DSM criteria created from the MFQ items with an “or” rule (see Table S2), while blue nodes are symptoms not contemplated by the DSM.

# Supplementary Figure S5: Expected influence centrality for the MFQ (n=1,070) with DSM and non-DSM features

Note. MFQ: Mood and Feelings Questionnaire. Gray nodes are DSM criteria created from the MFQ items with an “or” rule (see Table S2), while blue nodes are symptoms not contemplated by the DSM. On the Y-Axis, MFQ items ordered by highest to lowest expected influence centrality; on the X-Axis are z-standardized expected influence centrality values with zero as the mean value.

# Supplementary Figure S6. Network structure of PHQ-A items (A) and DSM items derived from the MFQ with an “or” rule (B; see Table S1 for a full explanation)

**
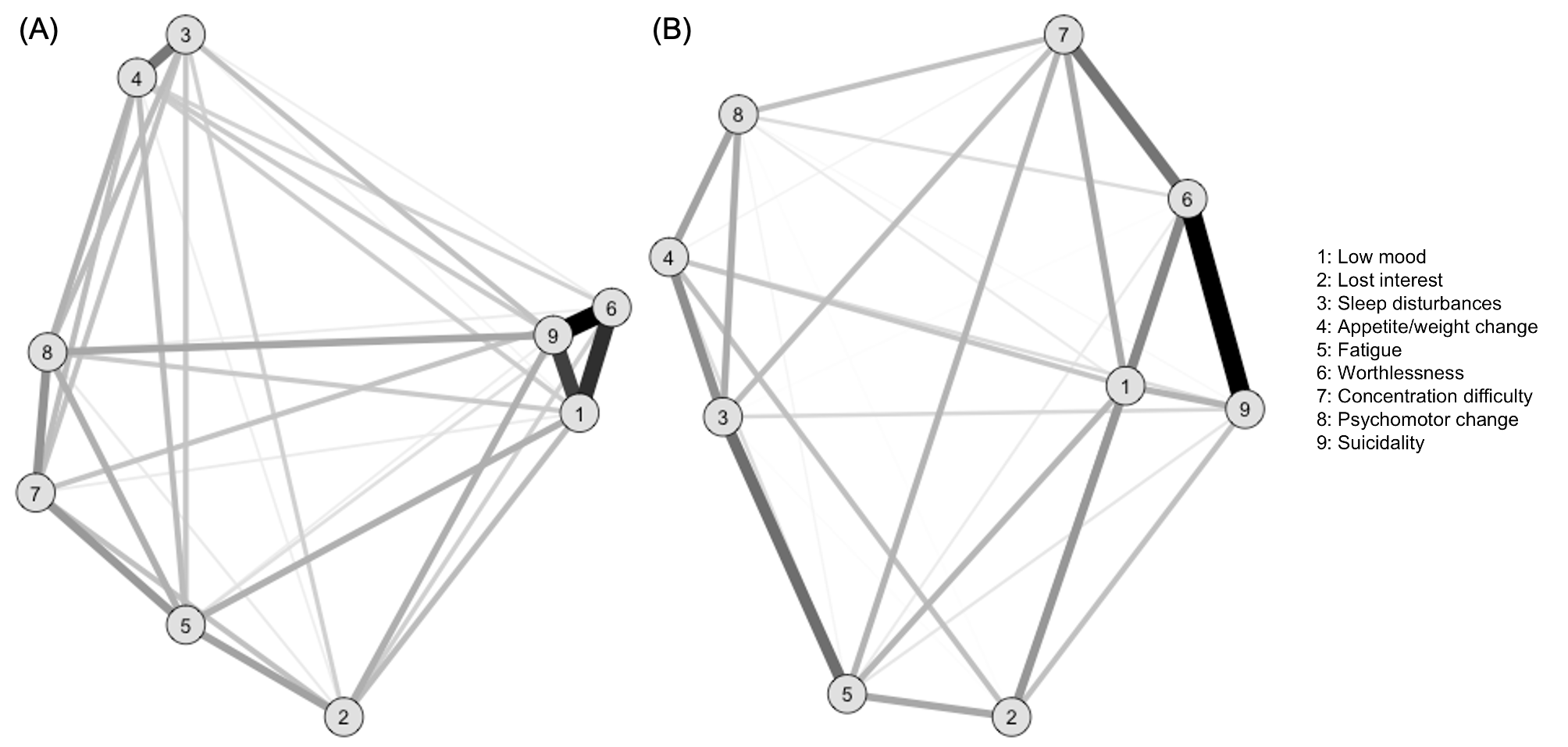
**

Note. PHQ-A: Patient Health Questionnaire – Adolescent Version. MFQ: Mood and Feelings Questionnaire. Lines represent positive associations. Line thickness and saturation represent correlation magnitude. Both Figure S6.A and S6.B’s layout are based on multidimensional scaling. Network comparison test (NCT) between the two graphs showed graphs to have different overall structures (i.e. connections between the nine DSM items change from the PHQ-A sample to the MFQ sample; S=0.128, p=0.01) although there was no difference in total connectivity (i.e. partial correlations were not larger in one graph than another; M=0.058, p=0.282).

# Supplementary Figure S7. Expected influence centrality of PHQ-A items and DSM items derived from the MFQ with an “or” rule


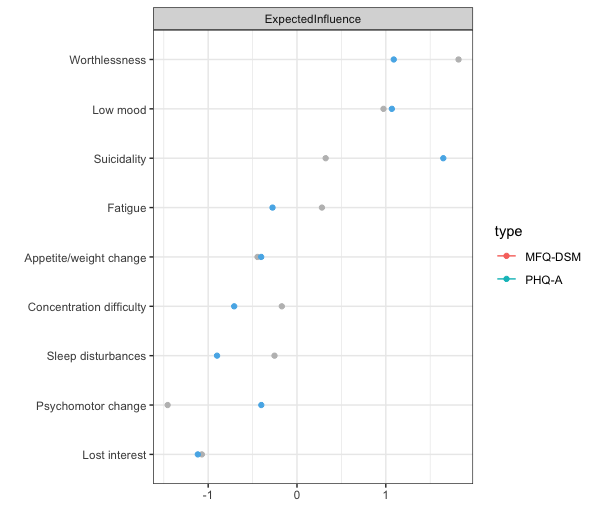


Note. PHQ-A: Patient Health Questionnaire – Adolescent Version. MFQ: Mood and Feelings Questionnaire. Gray points represent PHQ-A item centrality estimates; blue points represent DSM criteria derived from the MFQ items. On the Y-Axis, MFQ items ordered by highest to lowest expected influence centrality; on the X-Axis are z-standardized expected influence centrality values with zero as the mean values.

# Supplementary Figure S8. Expected influence centrality of males (blue) and females (red) from the MFQ network

**
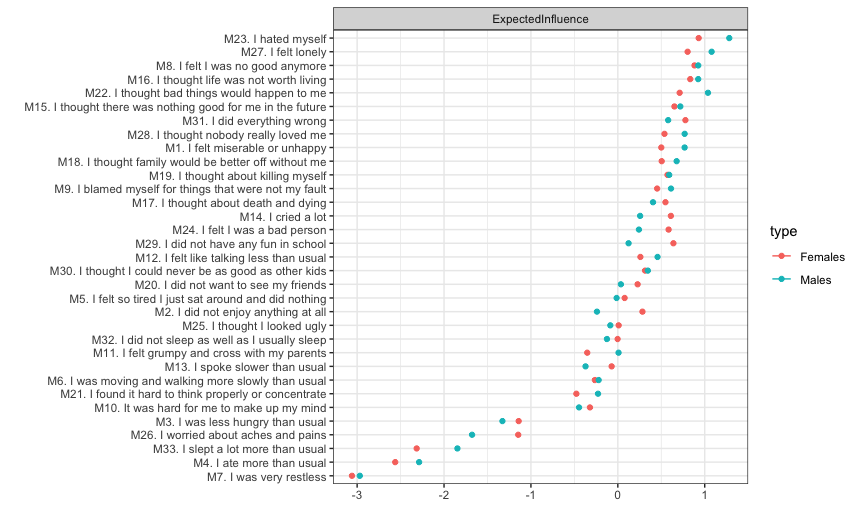
**

Note. MFQ: Mood and Feelings Questionnaire. “I hated myself” was the most central item for boys and girls, followed by “I felt lonely” and “I thought bad things would happen to me” for males; “I felt I was no good anymore” and “I thought life was not worth living” was the most central item for females. On the Y-Axis, MFQ items ordered by highest to lowest expected influence centrality; on the X-Axis are z-standardized expected influence centrality values with zero as the mean values.

# REFERENCES

1. Caspi A, Sugden K, Moffitt TE, Taylor A, Craig IW, Harrington HL, et al. Influence of life stress on depression: Moderation by a polymorphism in the 5-HTT gene. Science. 2003;301:386–9.

2. Rocha TBM, Hutz MH, Salatino-Oliveira A, Genro JP, Polanczyk GV, Sato JR, et al. Gene-environment interaction in youth depression: Replication of the 5-HTTLPR moderation in a diverse setting. American Journal of Psychiatry. 2015;172:978–85.

3. Kendler KS, Aggen SH, Flint J, Borsboom D, Fried EI. The centrality of DSM and non-DSM depressive symptoms in Han Chinese women with major depression. Journal of Affective Disorders. 2018;227:739–44.

4. Fried EI, Epskamp S, Nesse RM, Tuerlinckx F, Borsboom D. What are “good” depression symptoms? Comparing the centrality of DSM and non-DSM symptoms of depression in a network analysis. Journal of Affective Disorders. 2016;189:314–20.

5. Kendler KS. The Phenomenology of Major Depression and the Representativeness and Nature of DSM Criteria. American Journal of Psychiatry. American Psychiatric Publishing; 2016;173:771–80.
